# Survival of Viral Biowarfare Agents in Disinfected Waters

**DOI:** 10.1155/2010/412694

**Published:** 2010-12-16

**Authors:** Mary Margaret Wade, Amanda E. Chambers, Joseph M. Insalaco, Alan W. Zulich

**Affiliations:** ^1^Edgewood Chemical Biological Center, U.S. Army, RDECOM, Edgewood Area, Aberdeen Proving Ground, Aberdeen, MD 21010, USA; ^2^Science Applications International Corporation, P.O. Box 68, Gunpowder, Branch, Aberdeen, MD 21010, USA

## Abstract

Protecting civilian and military water supplies has received more attention since the United States began its war on terror in 2001. Both chlorine and bromine are used by branches of the U.S. military for disinfecting water supplies; however, limited data exists as to the effectiveness of these additives when used against viral biowarfare agents. The present study sought to evaluate the survival of selected viral biothreat agents in disinfected water. Disinfected water samples were spiked with vaccinia virus strain WR and Venezuelan equine encephalitis (VEE) virus strain TC-83 each separately to a final concentration of approximately 1 × 10^6^ PFU/mL, and survival was assessed by plaque assay. Both viruses were inactivated by 1 mg/L free available chlorine (FAC) and 2mg/L total bromine within one hour. In conclusion, these results demonstrate that both chlorine and bromine are effective disinfectants against vaccinia virus and VEE strain TC-83 at the concentrations tested.

## 1. Introduction

Since the tragic events of 2001, concern among many government agencies has risen with regard to protecting the nation's critical water infrastructure. And although chlorination is commonly used in the U.S. for disinfecting drinking water [1, 2], little data currently exits with regard to the persistence of biowarfare agents in chlorinated water, particularly viral biowarfare agents. Studies have been performed examining chlorine inactivation of bacterial biothreat agents [3–5], but few studies have examined free chlorine inactivation of viral biothreat agents, such as those that cause viral hemorrhagic fevers, viral encephalitis, or smallpox. One study examining the survival of the vaccine strain of Venezuelan equine encephalitis (VEE) virus in liquids reported that VEE strain TC-83 was reduced by more than 5 log _10_ within the 21-days in tap water with chlorine (between 4 and 5 mg/L), yet survived in distilled-deionized water at 4°C for the duration of the 21-day study [6]. 

Many branches of the armed services use chlorine to disinfect water; however, the U.S. Navy often utilizes bromine onboard ships for the same purpose and even less is known about the effectiveness of bromine against biothreat agents. Therefore, in the present study the U.S. Army Edgewood Chemical Biological Center (ECBC) sought to investigate the survival of selected viral biowarfare agents in waters representative of those used by branches of the military to include both chlorinated and brominated water using military relevant concentrations of each disinfectant. Smallpox and VEE are both considered potential bioterrorism agents and as such pose a high risk to national security. Both can be easily to moderately disseminated or transmitted and result in high to moderate mortality rates. Therefore, it was desired to investigate survival of these particular potential agents of bioterrorism in disinfected waters. Specifically, vaccinia virus (Smallpox surrogate) and VEE virus strain TC-83, which are avirulent representatives of the Category A and Category B agents, respectively, were employed in the present study.

## 2. Materials and Methods

### 2.1. Formulated Tap Water Preparation

Concentrated stock solutions were prepared using ASTM Type I deionized water and were stored at 4°C with a shelf life of 6 months (see Table 1 for list of stock solutions). Once stock solutions were prepared, approximately 500 mL of ASTM Type I deionized water was added to a 1-liter volumetric flask. Then each stock solution was added in the appropriate amount to achieve the desired final concentration (refer to Table 1) and the total volume was brought to 1 liter with deionized water. After 15–20 minutes, the pH was assessed and adjusted if needed (with minimal volume change) to 7.6–7.8. Once prepared, formulated tap water was stored at 4°C with a shelf life of one week.

### 2.2. Chlorine Stock Solution Preparation

1.00 gram of calcium hypochlorite (Logistics NSNno. 6840-00-255-0471, containing 65% available chlorine) was weighed and placed into a 22 mL glass vial. 20.0 mL of ASTM Type I deionized water was added, and the vial was capped and shaken vigorously for 1-2 minutes. The solids were allowed to settle for 5–10 minutes before using. The chlorine stock solution was used within eight hours.

### 2.3. Chlorine Addition

15 *μ*L of the chlorine stock solution (supernatant, not solids) was added to 100 mL of the formulated tap water. The free available chlorine (FAC) was measured using a Hach DR 2500 spectrophotometer per manufacturer's instructions (Hach Company, Loveland, CO). Chlorinated water was not considered stable and therefore was made fresh daily just prior to use. All glass bottles or vials were preconditioned with chlorinated water prior to the first use by soaking overnight. In addition, old chlorinated water was left in the bottle until a new batch was made in order to reduce the chlorine demand of the bottle surfaces.

### 2.4. Bromine Addition

A brominating cartridge (EVERPURE, P/N 255340-416, 1.25 kg of brominated resin, 30% bromine) was opened and approximately 25 mL of resin was transferred to a 500 mL, 0.20 *μ*m filter unit (Corning Life Science, Lowell, MA). Formulated tap water was poured into the same filter unit, filtered and collected. The bromine resin was retained by the filter. Total residual bromine in the collected formulated tap water was measured using a Hach DR 2500 spectrophotometer per manufacturer's instructions (Hach Company Loveland, CO). The total residual bromine level was adjusted if needed to achieve the desired final concentration of 2 mg/L by either diluting the sample with formulated tap water or passing (refiltering) the sample back over the bromine resin. Brominated water was not considered stable and therefore was made fresh daily just prior to use.

### 2.5. Cell Lines and Virus Source

Vero cells (CCL-81), BSC-40 cells (CRL-2761), and BHK-21 cells (CCL-10) were obtained from ATCC (Manassas, VA). Vaccinia virus strain WR and Venezuelan Equine Encephalitis virus strain TC-83 (VEE TC-83) were also obtained from ATCC. Vaccinia virus served as a biosafety level 2 surrogate for Variola major (smallpox) and the vaccine strain of VEE (TC-83) also served as a surrogate for virulent VEE. All cells were grown at 37°C with 5% CO_2_ in Dulbecco's Modified Eagle's Medium (DMEM) containing 10% heat-inactivated fetal bovine serum (FBS). Cells were passed on a twice weekly basis, and media were replaced daily.

### 2.6. Vaccinia Propagation

Vaccinia virus was propagated in BHK-21 monolayer cultures at 37°C. Cells were infected with virus for 1 hour in DMEM at 37°C. Following the absorption period, media were removed and replaced with fresh DMEM containing 10% FBS. Infected cells were harvested 48 hours postinfection and centrifuged at 650 × g at 4°C for 10 minutes. Pellets were resuspended in cell culture medium, freeze-thawed for three cycles, sonicated for four minutes on ice, and centrifuged at 650 × g at 4°C for 10 minutes. The supernatant served as the source for virus. The resulting virus served as the source of vaccinia for all experiments described below.

### 2.7. VEE Propagation

VEE strain TC-83 virus was propagated in Vero monolayer cultures at 37°C. Cells were infected with virus for one hour in DMEM at 37°C. Following the absorption period, media were removed and replaced with DMEM containing 10% FBS at 37°C. Infected cells were harvested and centrifuged at 650 × g at 4°C for 10 minutes. The supernatant was saved and stored at −80°C and served as the source of VEE strain TC-83 for all experiments described below.

### 2.8. Plaque Assays

BSC-40 cells (host of vaccinia virus) or Vero cells (host of VEE TC-83) were plated at a density of 3.0 × 10^5^ cells per well in twelve-well plates. Cells were allowed to reach confluence overnight at 37°C in DMEM containing 10% FBS. Prior to dilution, the vaccinia stock was sonicated on ice for 30 seconds. The virus was serially diluted from 1 : 10^2^ to 1 : 10^9^ in DMEM. The medium was removed from the cells, and the diluted virus was added. Virus absorption was for one hour at 37°C with occasional shaking. Following absorption, medium was removed and replaced with minimal essential medium (MEM) containing 5% FBS and 1% SeaPlaque Agarose. Forty-eight hours after infection, the cells were fixed in 7% formaldehyde for one hour. The agarose layer was removed, and cells were fixed for an additional hour in 7% formaldehyde. After removing the formaldehyde, plaques were visualized by staining with 0.01% crystal violet for 30 minutes. The plaques were counted, and the results were reported in plaque forming units per milliliter (pfu/mL).

### 2.9. Inoculation, Incubation, and Sampling

Survival of vaccinia virus strain WR and VEE TC-83 in chlorinated and brominated water (preparation of each described above) was determined by inoculating each water matrix with virus to a final concentration of approximately 1 × 10^6^ PFU/mL. At various time intervals, 1 mL of the spiked water sample was removed and sodium thiosulfate was added to a final concentration of 0.005% to quench any remaining disinfectant (Sigma Chemical Company, St. Louis, MO). The samples were serial diluted, and plaque assay was performed as described above. All water matrices tested were filter sterilized prior to inoculation. All samples were incubated at room temperature (21°C) after inoculation, and each virus was tested separately in each water. Military relevant time points were selected per guidance from the funding agency.

## 3. Results

**Figure 1:**
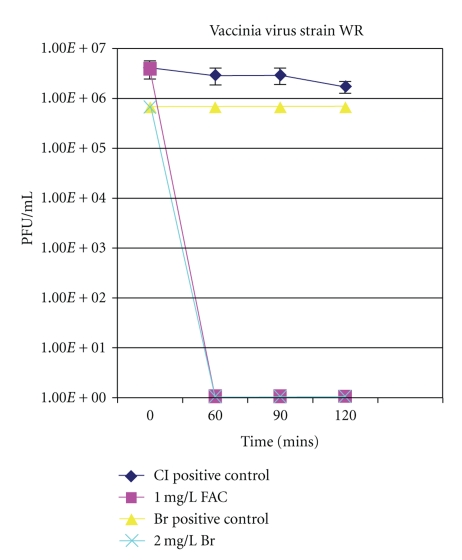
Survival of vaccinia virus strain WR in formulated tap water with 1 mg/L FAC and 2 mg/L total bromine. Positive control samples consisted of formulated tap water without disinfectant. All samples were incubated at 21°C, and, at intervals, viral titers were determined as described in Materials and Methods.

**Figure 2:**
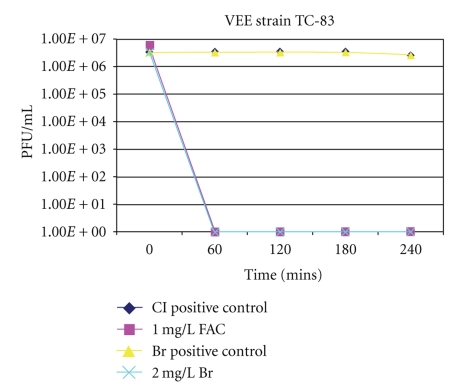
Survival of VEE strain TC-83 in formulated tap water with 1 mg/L FAC and 2 mg/L total bromine. Positive control samples consisted of formulated tap water without disinfectant. All samples were incubated at 21°C, and, at intervals, viral titers were determined as described in Materials and Methods.

Survival of vaccinia virus strain WR and VEE strain TC-83 in both chlorinated and brominated water over time is presented in Figures 1 and 2, respectively. All data is presented as the number of PFU/mL recovered over time by plaque assay. As shown in Figures 1 and 2, both viruses persisted in positive control samples, which consisted of formulated tap water without disinfectant, with no decrease in viability for the time points tested. However, in the presence of 1 mg/L FAC and 2 mg/L total bromine, neither virus was infectious at the earliest time point tested of one hour or at any subsequent time points tested.

## 4. Discussion

Although disinfection of water supplies is common practice in the U.S., limited data is currently available with regard to the length of time viral biothreat agents can survive in those waters. Both chlorination and bromination of water are practiced by branches of the military and therefore both were included at military relevant concentrations in the present study in order to ascertain their effectiveness as disinfectants against selected viruses. Survival of vaccinia virus strain WR and VEE strain TC-83 in formulated tap water with bromine or chlorine was monitored over time at room temperature using plaque assay to assess infectivity of the virus. Both disinfectants proved to be effective sanitizers against the viral biowarfare agents tested in a minimal amount of time making this study one of the first to report the survival of viral biothreat agents in chlorinated, and, moreover, brominated water. 

The U.S. Army and other government entities, in an effort to protect national and military water supplies, are placing great emphasis on developing rapid detection and identification technologies for biological agents in water. However, based on data provided in this study, monitoring chlorine or bromine levels in water supplies to ensure that adequate levels of disinfectant are present could prove sufficient for maintaining safe water supplies. Additional testing with a greater number of agents will be required before this decision can be made, and these tests are already underway to evaluate whether additional agents are easily killed by chlorination and bromination.

## Figures and Tables

**Table 1:** Preparation of formulated tap water.

Chemical	Concentration of Stock (mg/liter)	Amount of Stock added (mL)	Final concentration (mg/liter)
NaHCO_3_	10,000	10.0	100
MgSO_4_·7H_2_O	1,000	13.4	13.4
K_2_HPO_4_	1,000	0.7	0.700
KH_2_PO_4_	1,000	0.3	0.300
(NH_4_)_2_SO_4_	100	0.1	0.0100
NaCl	100	0.1	0.0100
FeSO_4_·7H_2_O	10.0	0.1	0.001
NaNO_3_	1,000	1.0	1.00
CaSO_4_	1,000	27.0	27.0
Humic acid^a^	1,000	1.0	1.00
Fulvic acid^b^	1,000	1.0	1.00

(a) IHSS Suwannee River Humic Acid Standard, Cat. No. 1S101H.

(b) IHSS Suwannee River Fulvic Acid Standard, Cat. No. 1S101F.
